# Supplementary material for: Characterizing the Countrywide Epidemic Spread of Influenza A(H1N1)pdm09 Virus in Kenya between 2009 and 2018
Source: Viruses. 2021 Sep 29;13(10):1956. doi: 10.3390/v13101956 (PMC8539974; doi:10.3390/v13101956)
Supplement: Supplementary file 1 [file viruses-13-01956-s001.zip › viruses-1340656-supplementary.pdf]

Supplementary Materials

Collation of contemporaneous global sequence dataset. Global comparison datasets for influenza A(H1N1)pdm09 virus were retrieved from the GISAID EpiFlu™ database (https://platform.gisaid.org/epi3/cfrontend; accessed on 19 January 2020). The datasets were prepared to determine the relatedness of the viruses in this report to those circulating around the world thus understand their global context. Only sequences with complete coding sequences sampled between March 2009 and December 2018 were included to improve the phylogenetic resolution of the analyses. The data were organized into a Microsoft Excel database which also stored the associated metadata (country of origin, date of isolation, subtype, and sequence length per segment). In-house python scripts were used in the extraction and manipulation of the data. Additionally, sequences were binned by calendar year for temporal analysis. A final dataset of 1,587 global sequences sampled between April 2009 and December 2018 was available (numbers in parenthesis indicate number of sequences): Africa (155); Asia (372); Europe (326); North America (356); South America (181); and Oceania (197). The accession numbers for the global dataset are available in the GISAID acknowledgement table in the report's GitHub repository, https://github.com/DCollinsOwuor/H1N1pdm09_Kenya_Phylodynamics/tree/main/Data/; accessed on 11 March 2021.

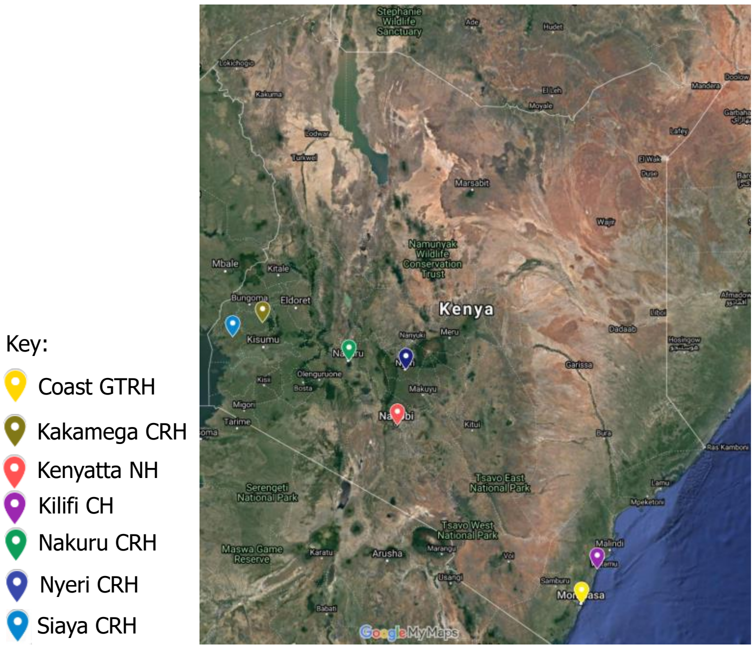

Figure S1. Map of Kenya showing the influenza sentinel surveillance sites for SARI used in this study. SARI, Severe Acute Respiratory Illness; GTRH, General Teaching and Referral Hospital; CH, County Hospital; CRH, County and Referral Hospital; NH, National Hospital.

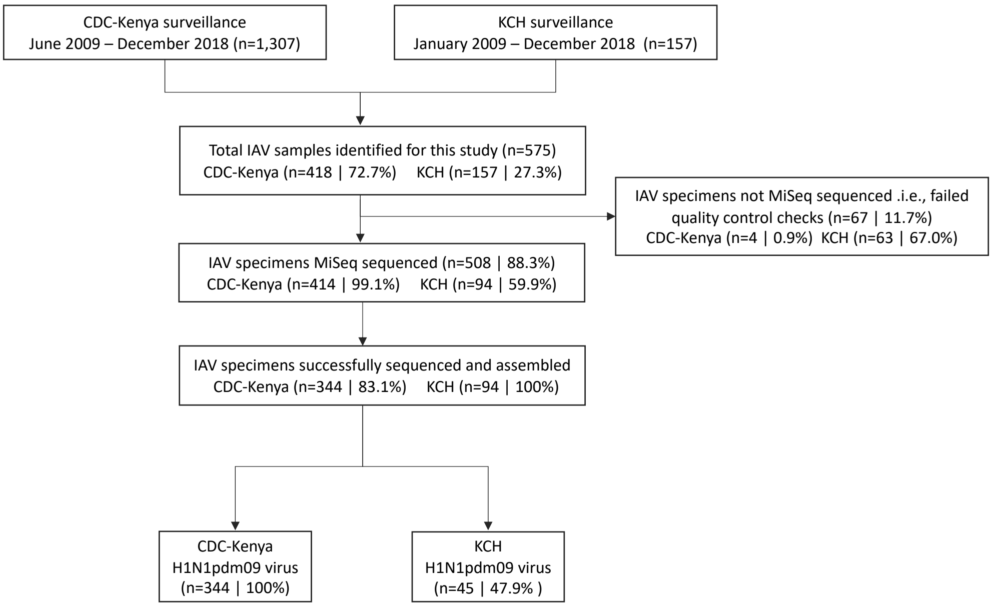

Figure S2. Sample processing flow for CDC-Kenya and KCH surveillance of IAV positive specimens, 2009-2018. Next generation sequencing generated 344 and 39 codon-complete A(H1N1)pdm09 virus sequences from the CDC-Kenya and KCH surveillance studies, respectively, which were used for this report. CDC, Centers for Disease Control; KCH, Kilifi County Hospital; IAV, influenza A virus.
